# Analytical studies assessing the association between extreme precipitation or temperature and drinking water-related waterborne infections: a review

**DOI:** 10.1186/s12940-015-0014-y

**Published:** 2015-03-27

**Authors:** Bernardo R Guzman Herrador, Birgitte Freiesleben de Blasio, Emily MacDonald, Gordon Nichols, Bertrand Sudre, Line Vold, Jan C Semenza, Karin Nygård

**Affiliations:** Department of Infectious Disease Epidemiology, Norwegian Institute of Public Health, Oslo, Norway; Oslo Centre for Statistics and Epidemiology, Department of Biostatistics, Institute of Basic Medical Sciences, University of Oslo, Oslo, Norway; European Programme for Intervention Epidemiology Training (EPIET), European Centre for Disease Prevention and Control, Stockholm, Sweden; European Centre for Disease Prevention and Control, Stockholm, Sweden; Gastrointestinal, Emerging and Zoonotic Diseases Department, Public Health England, London, UK; Norwich Medical School, University of East Anglia, Norwich, UK; Department of Hygiene & Epidemiology, University of Thessaly, Thessaly, Greece

**Keywords:** Review, Precipitation, Rainfall, Temperature, Waterborne infection

## Abstract

Determining the role of weather in waterborne infections is a priority public health research issue as climate change is predicted to increase the frequency of extreme precipitation and temperature events. To document the current knowledge on this topic, we performed a literature review of analytical research studies that have combined epidemiological and meteorological data in order to analyze associations between extreme precipitation or temperature and waterborne disease.

A search of the databases Ovid MEDLINE, EMBASE, SCOPUS and Web of Science was conducted, using search terms related to waterborne infections and precipitation or temperature. Results were limited to studies published in English between January 2001 and December 2013.

Twenty-four articles were included in this review, predominantly from Asia and North-America. Four articles used waterborne outbreaks as study units, while the remaining articles used number of cases of waterborne infections. Results presented in the different articles were heterogeneous. Although most of the studies identified a positive association between increased precipitation or temperature and infection, there were several in which this association was not evidenced. A number of articles also identified an association between decreased precipitation and infections. This highlights the complex relationship between precipitation or temperature driven transmission and waterborne disease. We encourage researchers to conduct studies examining potential effect modifiers, such as the specific type of microorganism, geographical region, season, type of water supply, water source or water treatment, in order to assess how they modulate the relationship between heavy rain events or temperature and waterborne disease. Addressing these gaps is of primary importance in order to identify the areas where action is needed to minimize negative impact of climate change on health in the future.

## Background

Mechanisms through which extreme precipitation, both increased and decreased, can contribute to the occurrence of waterborne infections are well documented. Heavy precipitation events increase the likelihood of water supply contamination due to the risk of sewer overflows [1]. Aging water treatment and distribution systems are particularly susceptible to heavy precipitation events, increasing the vulnerability of the drinking water supply. On the other hand, low precipitation may contribute to waterborne infections by increasing the percentage of sewage effluent in rivers when rainfall decreases or by increasing risk of groundwater contamination when the water table drops. In addition, many infectious agents and their vector and reservoir cycles are sensitive to temperature conditions [2].

A considerable amount of research is being conducted to map and assess risks, vulnerabilities and the impact of climate change in waterborne disease [3-5]. A recently published review [6] identified waterborne outbreaks potentially linked to an extreme water-related weather event and assessed how the different types of extreme weather events impact the occurrence of waterborne disease. Authors concluded that improving the understanding of the effects that different extreme water-related weather events have on waterborne disease is an important step towards finding ways to mitigate the risks.

Both the World Health Organization (WHO) and the European Centre for Disease Prevention and Control (ECDC) have emphasized the need for strengthening partnerships between health and climate experts, to improve scientific evidence of the linkages between health and climate drivers [7,8]. Despite the abundance of meteorological and epidemiological registries and databases, these are often not linked, preventing a more comprehensive understanding of potential associations [8]. Other publications have also highlighted additional obstacles to data access for research related to climate and water [9], and claim a reprioritization of public health research to ensure that funding is dedicated to explicitly studying the effects of changes in climate variables on food- and waterborne diseases [10].

To document the available knowledge, we performed a literature review of analytical research studies that have combined epidemiological and meteorological data to assess associations between extreme precipitation or air temperature and waterborne infections. This will help to identify specific areas where more specific research on this topic is needed.

## Methods

### Search strategy

The keywords used for searching relevant articles included both general and specific terms related to water, waterborne infections and precipitation or temperature related conditions (Table 1). These three groups of keywords were combined. The search strategy was run in the medical databases Ovid MEDLINE and EMBASE and in the multidisciplinary databases SCOPUS and Web of Science. Titles and abstracts of publications were searched for keywords. In order to focus on the most relevant and recent research, the search was limited to studies involving humans published in English between January 2001 and December 2013. In addition, a snowballing technique was used to review the reference lists of selected studies to identify additional articles.

**Table 1:** **Keywords used for searching in the literature**

**Thematic areas**	Specific terms*
**Water source**	Water, water supply, groundwater, surface water, water purification, water disinfection, sewage
**Waterborne infection**	Waterborne, gastroenteritis, outbreak, campylobacteriosis, Escherichia coli, cholera, cryptosporiosis, hepatitis A, giardiasis, salmonellosis, shigellosis, norovirus, typhoid fever
**Weather conditions**	Climate, weather, precipitation, rain, rainfall, temperature, humidity, season, flood, drought, snow

*Terms in the same box were combined with “or” in the search. Terms in the different rows were combined with “and” in the search.

### Data extraction strategy

Two independent reviewers screened titles for relevance obtained after running the search strategy. In a second step, selected abstracts were screened using the inclusion and exclusion criteria specified in Table 2. The full text of relevant studies were retrieved and assessed for eligibility. A sample of ten articles was reviewed by two independent reviewers in order to determine what data should be extracted. Dummy tables were designed for this purpose.

**Table 2:** **Inclusion and exclusion criteria**

**Inclusion criteria**	Analytical research studies in which the main objective was
	To estimate the association between extreme precipitation or temperature and drinking water-related waterborne outbreaks or infections
**Exclusion criteria**	Study type:
	-Outbreak reports reporting a single outbreak event.
	-Pure discussion papers or reviews without specific statistical analysis and results presented.
	-Studies without statistical analysis of associations (i.e. surveys).
	Events presented:
	-Outbreaks or trends of food-borne and vector-borne outbreaks or infections
	-Study of environmental conditions other than precipitation or air temperature
	-Main route of transmission other than drinking water.
	-Estimation of the association between extreme precipitation or temperature and concentration of microorganisms in water, but without data on human illness presented in the paper.
	-Study of seasonality not related to weather or climate data.
**Search strategy limited to:**	Population: Humans
	Publication year: January 2001-December 2013
	Language: English

The following data were extracted from the articles and included in Tables 3 and 4: first author, publication year, location of study (continent, country or region), study period (in years), waterborne infection studied and data source, study objective, exposure variable studied (precipitation or/and temperature) and data source, analytical methods used, additional information (whether the study took into account in the analysis seasonality, water source, water treatment, or water supply involved), and main associations and conclusions found in the study. Articles were classified according to the study units used (outbreaks or cases of infection).

**Table 3:** **Region, study period, waterborne infections and data sources in the included articles by type of study unit**

**Study units**	**First author publication year**	**Continent**	**Country/Region**	**Study period**	**Waterbone disease under study**	**Waterborne disease Data source**
**Outbreaks**	Yang [12]; 2012	Global	-	1991-2008 (18 years)	Drinking water related waterborne disease outbreaks (+ other water-associated diseases)	Database developed by the Global Infectious Disease Epidemiology Network (GIDEON)
	Curriero [14]; 2001	North America	United States	1948-1994 (47 years)	Drinking water related waterborne disease outbreaks with contamination at the water source	Surveillance data at national level
	Thomas [11]; 2006	North America	Canada	1975-2001 (27 years)	Drinking water related waterborne disease outbreaks	Published compilation at national level
	Nichols [13]; 2009	Europe	England and Wales	1910-1999 (90 years)	Drinking water related waterborne disease outbreaks	Medline search, published papers and unpublished reports
**Cases of infection**	Tornevi [22]; 2013	Europe	Gothenburg, Sweden	2007-2011 (5 years)	Telephone calls to acute gastrointestinal illnesses	Nurse advice line
	Louis [18]; 2005	Europe	England and Wales	1990-1999 (10 years)	Campylobacteriosis cases	Surveillance data at national level
	Eisenberg [15]; 2013	Central America	Haiti	2010-2011	Cholera cases	Registry at a hospital
						Internally displaced person camp data
						Reports at the ministry
	White [25]; 2009	North America	Philadelphia, United States	1994-2007 (14 years)	Campylobacteriosis cases	Surveillance data at national level
	Drayna [26]; 2010	North America	Wisconsin, United States	2002-2007 (6 years)	Physician visits of gastrointestinal infections/diarrhea	Administrative records
	Teschke [21]; 2010	North America	Vancouver, Canada	1995-2003 (9 years)	Physician visits and hospitalization records of various gastrointestinal diseases with potential to be waterborne	Administrative records
	Harper [16]; 2011	North America	Nunatsiavut, Canada	2005-2008 (4 years)	Gastrointestinal illness related visits	Administrative records
	Hashizume [27]; 2007	Asia	Dhaka, Bangladesh	1996-2002 (7 years)	Weekly number of patients visiting a hospital due to non-cholera diarrhea	Administrative records
	Vollaard [23]; 2004	Asia	Jakarta, Indonesia	2001-2003 (3 years)	Typhoid or paratyphoid fever cases	Consultations at hospitals and outpatient health centers
	Kelly-Hope [33]; 2007	Asia	Vietnam	1991-2001 (11 years)	Shigellosis, cholera and typhoid fever cases	Surveillance data at national level and published papers and unpublished reports
	Emch [31]; 2008	Asia	-Hue and Nha Tranng, Vietnam	−1985-2003 (23 years)	Cholera cases	Records from a research centre/surveillance data at national level
			-Matlab,Bangladesh	−1983-2003 (21 years)		
	Constantin de Magny [30]; 2008	Asia	-Kolkata, India	1997-2006(10 years)	Cholera cases	Administrative records
			-Matlab, Bangladesh			
						Records from a research center
	Wang [24]; 2012	Asia	Guizhou, China	1984-2007 (24 years)	Typhoid and paratyphoid fever cases	Surveillance data at national level
	Chen [29]; 2012	Asia	Taiwan	1994-2008 (15 years)	Hepatitis A, enteroviruses, shigellosis cases	Surveillance data at national level
	Jutla,[32]; 2013	Asia and Central America	-Northern India and Pakistan	−1875-1900 (26 years)	Cholera cases	Reports from the Government and previous published data
			-Haiti	-2010		
	Singh [20]; 2001	Oceania and Australia	Pacific Islands	1978-1998, with two missing years(19 years)	Diarrhea cases	Surveillance data at national level
	Hu [17]; 2007	Oceania and Australia	Brisbane, Australia	1996-2004 (9 years)	Cryptosporidiosis cases	Surveillance data from the regional level
	Rind [34]; 2010	Oceania and Australia	New Zealand	1997-2005 (9 years)	Campylobacteriosis cases	Surveillance data at national level
	Britton [28]; 2010	Oceania and Australia	New Zealand	1997-2006 (10 years)	Cryptosporidiosis and Giardiasis cases	Surveillance data at national level
	Sasaki [19]; 2009	Africa	Lusaka, Zambia	2003-2004; 2005-2006	Cholera cases	Records at a treatment centre

Literature Review (n = 24).

**Table 4:** **Region, objective, exposure variables and data sources, analytical method, results and conclusions in the included articles by type of study unit**

**Study units**	**First author publication year**	**Objective**	**Exposure variable under study (Precipitation/Air temperature)**	**Exposure variable data source**	**Analytical method**	**Additional information**	**Association found**
**Outbreaks**	Yang [12]; 2012	Risk factors associated with spatio-temporal distributions of water-associated outbreaks	Average precipitation per year	Records from international organizations	Zero-inflated Poisson regression	-	Waterborne diseases are inversely related to average annual precipitation.
			Global average accumulated temperature (degree-days)				
							No association between temperature and waterborne disease.
	Curriero [14]; 2001	Association between extreme precipitation and waterborne disease outbreaks.	Extreme precipitation above certain threshold by watershed	Readings of relevant weather stations	Monte Carlo version of the Fisher exact test	Analysis stratified by water source and control for seasonality	Positive association between extreme precipitation and outbreak occurrence
							Both for surface water (strongest association during the month of the outbreak) and groundwater contamination (2-month prior to the outbreaks)
	Thomas [11]; 2006	Test the association between high impact weather event and waterborne disease outbreaks	Accumulated precipitation, smoothed using a five-day moving average, maximum percentile of the accumulated precipitation amount, number of days between the maximum percentile and the case or control onset day temperature	Readings of relevant weather stations	Time-stratified matched case-crossover analysis	Control for seasonality	Positive association between accumulated precipitation percentile and outbreak occurrence
							Positive association between degree-days above 0 C and outbreak occurrence
			Degree-days above 0 C, the maximum temperature smoothed using a five-day moving average, and the number of days between max temp and the case and the control onset day				
	Nichols [13]; 2009	Association between precipitation and outbreaks of drinking water related disease.	Cumulative precipitation in four time periods prior to each outbreak	Readings of relevant weather stations	Time-stratified matched case-crossover analysis	Water source, season, water supply considered as effect modifiers	Positive association with excess precipitation over the previous week and low precipitation in the three weeks before the week of the outbreak.
			Excessive precipitation: total number of days in which the precipitation exceeded a certain upper limit				
							Greater risk in groundwater, spring and private water supplies. These interactions were non-significant when including them together in a model, suggesting confounding.
**Cases of infection**	Tornevi [22]; 2013	Determine if variation in the incidence of acute gastrointestinal illnesses is associated with upstream precipitation	Daily precipitation	Readings of relevant weather stations	Poisson regression (with nonlinear distributed lag function)	Control for seasonality	Heavy precipitation was associated with increased calls.
	Louis [18]; 2005	Investigate the relationship between environmental conditions and *Campylobacter* infections	Precipitation divided into three categories up and down a certain threshold	Readings of relevant weather stations	Time series analysis	Seasonality and water supply also included in the study	*Campylobacter* rates were correlated with temperature
					Linear regression		
							No association with precipitation
							No association with surface water.
			Daily max and minimum temperature				
	Eisenberg [15]; 2013	Examine the relationship between cholera and precipitation in Haiti including statistical and dynamic models	Cumulative daily totals for precipitation	Rain gauges and satellite measurements	Statistical modeling	Control for seasonality	All analysis support a strong positive association between precipitation and cholera incidence in Haiti
					Quasi-Poisson regression (with nonlinear distributed lag function)		
					Granger Causality Wald Test		
					Case-crossover analysis		
					Dynamic modeling		
	White [25]; 2009	Association between environmental factors and campylobacter infection	Precipitation	Readings of relevant weather stations	Poisson regression	Control for seasonality	Weekly incidence was associated with increasing mean temperature.
			Temperature				
					Time-stratified matched case-crossover analysis		
							No association with precipitation
	Drayna [26]; 2010	Association between precipitation and acute gastrointestinal illness in pediatric population	Total daily precipitation, extreme considered above a certain percentile	Readings of relevant weather stations	Autoregressive moving average (ARMA) model	Control for seasonality	Positive association between precipitation and daily visits
	Teschke [21]; 2010	Association between the incidence of intestinal infections and environmental factors	Precipitation categories according accumulated millimeters of rain over certain periods	Readings of relevant weather stations	Logistic regression	Season, water supply, water source, disinfection and well depth included as variables	The association between incidence of disease and precipitation did not remain when controlling for other variables
							Water chlorination was associated with reduced physician visits
							Two water systems with the highest proportion of surface water had increased incidence
							Private well water and well depth were not associated with increased risk
	Harper; [16]; 2011	Association between weather variables and gastrointestinal-related clinic visits	Total daily precipitation	Readings of relevant weather stations	Zero-inflated Poisson regression	Control for seasonality	Positive associations were observed between high levels of water volume input (precipitation + snowmelt) and IGI clinic visits.
			Daily average temperature				
							No association with temperature
	Hashizume [27]; 2007	Impact of precipitation and temperature on the number of non-cholera diarrhea cases	Daily Precipitation, weekly means Above/below certain threshold	Records from national level	Poisson regression	Control for seasonality	Non-cholera diarrhea cases increased both above and below a threshold level with high and low precipitation in the preceding weeks. Cases also increased with higher temperature.
			Daily minimum/maximum temperature, weekly means				
	Vollaard [23]; 2004	Determine risk factors for typhoid and paratyphoid fever in an endemic area	Precipitation	Interviews with the participants	Logistic regression	-	Flooding was associated with the occurrence of paratyphoid fever. Flooding was not associated with typhoid fever.
			Flooding: defined as inundation of the house of a participant in the 12 months preceding the investigation				
	Kelly-Hope [33]; 2007	Environmental risk factors of cholera, shigellosis and typhoid fever infections	Precipitation	Worldwide maps generated by the interpolation of information from ground-based weather stations	Linear regression	Type of water supply	Shigellosis and cholera were positively associated with precipitation
			Temperature				
							Typhoid fever was not associated with precipitation
							No association with temperature
	Emch [31]; 2008	Association between cholera and the local environment	Monthly precipitation	Readings of relevant weather stations	Ordered probit model to analyze ordinal outcome (Bangladesh). Probit model for dichotomous outcome. (Vietnam).	-	Temperature and precipitation not associated with cholera
			Monthly temperature				
	Constantin de Magny [30]; 2008	Association of environmental signatures with cholera epidemics	Monthly precipitation	Merged satellite/gauge estimates	Quasi Poisson regression	Control for seasonality	Positive association between cholera and increased precipitation in Kolkata.
							No association cholera and increased precipitation in Matlab
	Wang [24]; 2012	Impact of meteorological variations on para/typhoid fever (PTF)	Monthly cumulative precipitation	Records from national level	-Spearman’s rank correlation analysis to analyze the association between the infection incidence and the weather variables	-	Temperature and precipitation were positively associated with the monthly incidence of PTF
					Wavelet analysis and wavelet coherence to detect the variation of periodicity over time		
			Monthly average temperature				
	Chen [29]; 2012	Association between precipitation and distribution patterns of various infectious diseases, including water-borne	Precipitation coded as: regular, torrential and extreme torrential	Readings of relevant weather stations	Poisson regression (with GAM and GAMM)	Control for seasonality using monthly indicator	Daily extreme precipitation levels correlated with the infections
	Jutla, [32]; 2013	Seek an understanding between hydro-climatological processes and cholera in epidemic regions	Precipitation and temperature above/below average during the previous months	Reports from the government	Spearman’s rank correlation analysis	-	India. -Odds of cholera occurring were significantly higher when the temperature was above climatological average over the previous two months. Odds of cholera outbreak was higher when above average precipitation occurs.
				satellite sensors			
			Daily precipitation and temperature				
							Haiti: Strong correlation between precipitation and cholera cases.
	Singh [20]; 2001	Association between climate variability and incidence of diarrhea	Precipitation : dichotomous variable above/below certain threshold	Gridded data from international institute	Linear regression Poisson	Control for seasonality	Positive association between annual average temperature and rates of diarrhea
							Extremes of precipitation were independently associated with increased reports of diarrhea
			Annual average temperature				
					regression		
	Hu [17]; 2007	Impact of weather variability on the transmission of cryptosporidiosis.	Monthly total precipitation	Records from national level	Poisson regression	Control for seasonality	Association between cryptosporidiosis and monthly maximum. temperature
					Seasonal auto-regression integrated moving average (SARIMA)		
		Explore the difference in the predictive ability between Poisson regression and SARIMA models	Monthly mean minimum/maximum temperature				
	Rind [34]; 2010	Association between climate factors and local differences in campylobacteriosis rates	Monthly mean maximum total precipitation	Records from research center	Linear regression	Water supply, seasonality	No association found between temperature and precipitation and campylobacteriosis rates
			Monthly mean maximum daily temperatures				
	Britton [28]; 2010	Association between precipitation and ambient temperature and notifications of cryptosporidiosis and giardiasis	Average annual precipitation to evaporation ratio	Mathematical surfaces fitted to long run average climate station data	Negative binomial regression	Water supply	Giardiasis: positive association between precipitation and temperature.
							Cryptosporidiosis: positive association with precipitation and negative association with temperature. The effect of precipitation was modified by the quality of the domestic water supply
			Average annual temperature				
	Sasaki [19]; 2009	Association between precipitation patterns and cholera outbreaks.	Daily precipitation data	Records from national level and readings of relevant weather stations	Spearman rank correlation analysis		Increased precipitation was associated with the occurrence of cholera outbreaks

Literature Review (n = 24).

## Results

**Figure 1:**
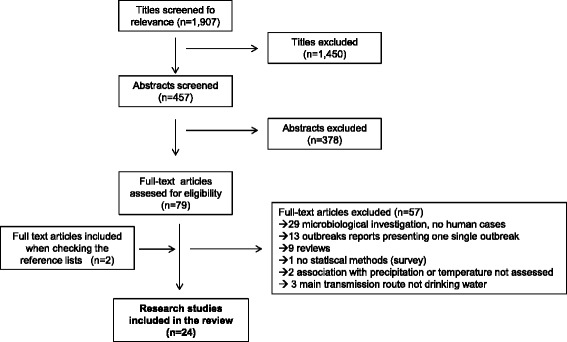
**Article selection strategy.**

Once duplicates were removed, a total of 1907 titles were obtained using the initial search terms. Following screening of titles, results were limited to 457 articles. After screening abstracts for relevance, 79 full-text articles were read full text, of which 57 were excluded. Two articles were included after checking the reference lists of the already selected articles. In total, 24 analytical research articles, in which the association between extreme precipitation or air temperature and waterborne infections had been assessed, were included in the literature review (Figure 1).

### Studies of drinking water-related waterborne infections, geographical location and data sources

#### Articles using outbreaks as study units (n = 4)

Four studies used drinking water related waterborne outbreaks as study units [11-14]. Two articles presented studies that were performed using data from North America (Canada and United States) [11,14] while one used data from Europe (England and Wales) [13]. One study included data from several continents [12]. There were different data sources used to obtain outbreak data, including surveillance data, publicly available databases, previous published compilations and unpublished reports. The four studies assessed the association between outbreaks and precipitation. Two of them also studied the relationship with temperature. Meteorological data under study were obtained from records available at international organizations or from readings from the relevant weather stations.

### Articles using cases of infection as study units (n = 20)

The remaining 20 articles used cases of infection as study units [15-34]. Most of the articles (n = 7) were performed in Asia (Bangladesh, Indonesia, Vietnam, India, Taiwan and China) [23,24,27,29-31,33]. Four were performed in North America (United States and Canada) [16,21,25,26], four in Oceania (Australia, New Zealand and Pacific Islands) [17,20,28,34], two in Europe (Sweden; and England and Wales) [18,22], one in central America (Haiti) [15], and one in Africa (Lusaka) [19]. One article used data from more than one continent, Asia and Central America [32].

The most common approach was to use cases of gastrointestinal infections without specifying the type of microorganism (n = 6). Among those studies focusing on specific microorganisms, cholera was most frequently studied (n = 6), followed by campylobacteriosis (n = 3) and typhoid fever (n = 3). Other infections, such as shigellosis, cryptosporidiosis, giardiasis, hepatitis A and paratyphoid fever, were also studied.

Cases of infection were obtained from several sources, including surveillance data, clinical records and registries, governmental reports and nurse advice telephone lines. All studies assessed the association between cases of infection and precipitation, while eleven of them also examined the relationship with temperature. The meteorological data under study were obtained from records available at international organizations, satellite sensors, gauge estimates, interviews or from local weather stations.

### Definition extreme precipitation or temperature, covariates and statistical analysis

The definition of extreme weather events varied across the studies. There were different ways of categorizing meteorological variables, according to the amount or range of precipitation (i.e. groups including different categories; accumulated; smoothed using a certain number of days moving average; dichotomous, above and below a threshold; total in a given period; exceeded the upper limit of a given reference range). Only seven articles presented analyses stratified by water source or type of water supply, aiming to disentangle differences in the association with the occurrence of waterborne infections.

Analysis using Poisson regression or other types of count model regression was the most commonly adopted method to investigate whether variation in disease occurrence could be partly explained by changes in variables related to extreme weather events. Count model regression was used in eleven studies, one with outbreaks [12] and ten with cases of infections [15-17,20,22,25,27-30]. In some cases, the Poisson regression model was adjusted to account for: a) overdispersion, either by estimating an additional dispersion parameter using quasi-Poisson regression models [15,30] or more formally by using negative binomial regression models [28], b) excess zero counts in the observations, by using Zero-inflated Poisson regression models [12,16]. Time series data are prone to be influenced by seasonal and long-term variations, which may mask the short-term association between disease and extreme weather events. Seasonal trend decomposition was conducted in different ways, such as by adding trend and seasonal components into the Poisson regression [17], or by using Fourier terms [20,25,27]. In some studies, temporal correlations were handled by using generalized additive models (GAM) with time and sometimes other variables related to weather were added as smoother variables [16,29]. Delayed effects and a time varying relationship between the exposure and outcome variables were considered using generalized additive mixed models (GAMM) [29] or nonlinear distributed lag functions [15,22]. Case-crossover analysis was most frequently used when the study units were outbreaks [11,13]. It was also used in two studies using cases of infections [15,25]. In this analysis, the weather exposure at the location of an outbreak was compared with the exposures at the same location and same time of the year during control periods without an outbreak through use of conditional logistic regression. The method controls for time-invariant seasonal and geographic differences by design, although it assumes that neither exposure nor confounders change in a systematic way over the course of the study.

### Findings of the studies

All four publications studying outbreaks found an association between precipitation and waterborne disease. Three found a positive association with extremes of precipitation [11,13,14], and one found an inverse association between waterborne outbreaks and average precipitation [12]. Among the two studies that assessed the association with temperature, one found a significant positive association [11]. Of the twenty articles using cases of waterborne infection as study units, amount of precipitation was found to have a positive association with infection in nine of them [15,16,19,22,24,26,28,29,32]. Two studies found a positive association in both extremes of precipitation (low and high) [20,27] and six did not find an association [17,18,21,25,31,34]. In three studies, statistically significant results were heterogeneous depending on the diseases or geographical regions they were assessing [23,30,33]. Regarding temperature, seven studies found a direct association between infections and temperature [17,18,20,24,25,27,32] and four did not find an statistical association [16,31,33,34]. In one study, statistically results depended on the disease that was being studied [28].

## Discussion

This review has identified twenty four analytical research studies in which epidemiological and meteorological data have been linked in order to assess associations between extreme precipitation or air temperature and waterborne outbreaks or cases of infection. The findings presented in the different articles are heterogeneous, highlighting the complex relationship between precipitation or temperature driven transmission and waterborne infections. Although most of the studies identified a positive association between increased precipitation or temperature and infection, there were several in which this association was not evidenced. A number of articles also identified an association between decreased precipitation and infections. Very few articles presented stratified analyses that took into account the type of water treatment, water source or water supply involved.

Although research on this topic has been performed in different continents, most of the studies were conducted in Asian countries. Only few articles have presented data from Europe or Africa and none presented results from South America, resulting in limited evidence-based information on the influence of extreme weather on waterborne infections in these regions. Most of the publications used cases of infection as study units and only four used outbreaks as units. Of those using cases of infection, cholera or cases of gastroenteritis without a specific etiology were the infections most frequently studied. A variety of study designs and statistical methods, mainly count model regressions and case-crossover analysis, were used.

Several limitations and challenges of the studies were stated by the authors of the reviewed studies. Underreporting is an inherent problem in surveillance systems, and with respect to waterborne outbreaks or infections, the notified cases likely represent just the tip of the iceberg of the true disease burden [35]. However, in terms of estimating the association between weather events and infections or outbreaks, underreporting would only be the cause of bias if reporting is correlated with weather variables [36]. There is lack of consensus about the definition of extreme precipitation or temperature. An association might be found more easily depending on the threshold level that was used to classify extreme precipitation or temperature events. The classification of an extreme weather event is a key issue and needs to be defined according to the regional meteorological pattern. In certain occasions, small data sets in terms of number of observations limit statistical power. One possible solution for sparse data is to aggregate explanatory and outcome variables by week, month or year. However, this may reduce the variation in the data and smooth the relationships with previous weather events. Extreme weather events generally occur on a local scale. This implies that the results obtained from analyzing national, regional or local level will be different and may have noticeable consequences for the interpretations. As an example, presenting results by census area unit instead of national level could allow for variation in exposure across a region or country, although this is not always possible due to limited availability of data. The optimal choice of time lag between weather event and occurrence of a given waterborne disease event is challenging, as these events generally do not occur simultaneously. Using the same time lag for all cases linked to specific weather events is not possible given the variation in incubation periods among and within different infections. Understanding all these issues is necessary in order to select the time lag most relevant for a given disease.

Our review has covered a period of 13 years and has used four different databases, two medical and two multidisciplinary, to identify potential relevant peer reviewed publications in a systematic way. Although relevant literature could have been missed for a number of reasons (not peer reviewed, published before 2001 or in other languages than English, not identified by our search terms, unpublished results), our results show that there is potential to generate more scientific evidence to better understand the association between extreme precipitation or air temperature and waterborne outbreaks or cases of infection.

## Conclusion

The heterogeneity of results presented in the articles identified in this review reflect the complexity of the relationship between extreme precipitation or air temperature and waterborne disease .There are several factors that could play a role on it, such as the specific type of microorganism, the geographical region, season, type of water supply, water source or water treatment. We encourage researchers to conduct studies examining these potential effect modifiers, in order to assess how they modulate the relationship between heavy rain events or temperature and disease. Addressing the gaps will be central for public health experts in order to identify the priority areas where action is needed to minimize negative impact on the health in future climate.

## Abbreviations

WHO: World Health Organization
ECDC: European Centre for Disease Prevention and Control
